# Correlation between hyperbilirubinemia risk and immune cell mitochondria parameters in neonates with jaundice

**DOI:** 10.3389/fped.2023.1200099

**Published:** 2023-06-16

**Authors:** Yingying Wang, Hongwei Wang, Qiang Zhang, Shanshan Li, Yiping Mao, Jiajin Lu, Yeqin Shen, Yaping Han

**Affiliations:** Shaoxing Keqiao Women & Children's Hospital, Shaoxing, Zhejiang, China

**Keywords:** bilirubin, mitochondrial mass, neonatal jaundice, single cell mitochondrial mass, T-Lymphocyte Subsets

## Abstract

**Purpose:**

To explore the correlation between mitochondria parameters of immune cells and hyperbilirubinemia risk in hospitalized neonates with jaundice.

**Methods:**

This retrospective study included jaundiced neonates born between September 2020 and March 2022 at Shaoxing Keqiao Women & Children's Hospital. The neonates were divided into low, intermediate-low, intermediate-high, and high-risk groups according to the hyperbilirubinemia risk. The purpose parameters including percentage, absolute count, mitochondrial mass (MM), and single-cell MM (SCMM) of peripheral blood T lymphocytes detected by flow cytometry were collected.

**Results:**

Finally, 162 neonates with jaundice (47, 41, 39, and 35 with low, intermediate-low, intermediate-high, and high-risk) were included. CD3^+^ SCMM was significantly higher in the high-risk group compared with the low and intermediate-low-risk groups (both *P *< 0.0083), CD4^+^ SCMM was significantly higher in the high-risk group compared with the three other groups (all *P *< 0.0083), and CD8^+^ SCMM was significantly higher in the intermediate-low and high-risk groups compared with the low-risk group (both *P *< 0.0083). CD3^+^ (*r* = 0.34, *P *< 0.001) and CD4^+^ (*r* = 0.20, *P *= 0.010) SCMM positively correlated with bilirubin levels.

**Conclusions:**

The mitochondrial SCMM parameters differed significantly among jaundiced neonates with different hyperbilirubinemia risks. CD3^+^ and CD4^+^ T cell SCMM values were positively correlated with the serum bilirubin levels, and might correlated with hyperbilirubinemia risk.

## 1. Introduction

Bilirubin is the main metabolite of iron porphyrin compounds. Serum bilirubin levels exceed 85 μmol/L in approximately 60% of term newborns and 80% of preterm infants in the first week after birth, and jaundice occurs (1). Most cases of neonatal jaundice are non-pathologic and may be due to physiologic reduction in bilirubin conjugating and excreting mechanisms and/or increased bilirubin production (2–5). Hyperbilirubinemia significant enough to require phototherapy has been reported in approximately 10% of term and 25% of near preterm infants (2–5). The pathologic causes of neonatal jaundice include hemolysis, enzyme deficiencies, or liver and biliary tract abnormalities (2–5).

Physiological concentrations of bilirubin can modulate intracellular signaling pathways involved in immunosuppression, prevent diseases associated with an increased oxidative stress (6), and produce anti-inflammatory effects by inhibiting NF-κB and the activation of inflammatory vesicles and regulate the morphology and function of mitochondria of immune cell (7), and mitochondria play central roles in the activation, differentiation, and survival of immune cells (8). Recently, serum bilirubin has been found to regulate dendritic cells, natural killer cells, and bone marrow-derived suppressor cells in peripheral blood and affect the composition type of the peripheral T cells (9). Excessive bilirubin can damage cell membranes, causing oxidative damage and disrupting cell signaling, leading to mitochondrial dysfunction (10), which can result in immune system disruption or hypoimmunity in affected children (9, 11) and even affect the integrity of the blood-brain barrier (12), causing irreversible neurological damage (12, 13).

For decades, the diagnosis and intervention monitoring of jaundice have relied on total serum bilirubin levels, despite their poor ability to predict the outcomes (13). Tests on immune cells, metabolic function, and other indicators could more objectively and accurately reflect the immune status of neonates with jaundice. Therefore, it could be hypothesized that tests evaluating the mitochondria of peripheral immune cells in newborns with jaundice might better reflect the prognosis and risks associated with excessive bilirubin.

Therefore, this retrospective study aimed to explore the correlation between mitochondria parameters of immune cells and bilirubin risk levels in hospitalized neonates with jaundice.

## 2. Patients and methods

### 2.1. Study design and patients

This retrospective analysis included jaundiced neonates born between September 2020 and March 2022 at Shaoxing Keqiao Women & Children's Hospital. This study was reviewed and approved by the Ethics Committee of Shaoxing Keqiao Women & Children's Hospital. The requirement for informed consent was waived by the committee because of the retrospective nature of the study.

The inclusion criteria were: (1) full-term newborns (gestational age ≥37 weeks) of <28 days of age and (2) diagnosed with neonatal jaundice based on Practical Neonatology, 5th edition (2019). The exclusion criteria were: (1) jaundice combined with other serious illnesses such as neonatal sepsis and intracranial hemorrhage in newborns, (2) incomplete information, or (3) blood samples for immune cell mitochondrial were taken after phototherapy. In particular, the subjects of our study were all from NICU children who were bottle fed by staff and have NO breastfeed.

Neonates were divided into low, intermediate-low, intermediate-high, and high-risk groups according to the hyperbilirubinemia risk, evaluated by Bhutani curves (14).

### 2.2. Data collection

The clinical characteristics of the included neonates with jaundice was collected, including day age, gestational age, sex, birth weight, serum bilirubin value (μmol/L), number of days in the hospital, clinical diagnosis, and immune cell mitochondria parameters. The immune cell mitochondrial testing is part of the routine immunoassays (flow cytometry) performed for neonatal jaundice, including CD3^+^, CD4^+^, and CD8^+^ cell percentages, absolute counts, mitochondrial mass (MM), and single-cell mitochondrial mass (SCMM). Sample preparation protocol: (a) monoclonal antibody preparation of pre-mixed reagents; (b) antibodies were mixed with peripheral blood samples, which were vortex mixed and incubated away from light; (c) add hemolysin for red blood cell lysis; (d) test by flow cytometer after mixing; (e) the flow cytometry data (.fcs) were calibrated by *the human lymphocyte mitochondrial function analysis system* (software) and then output to keep the accuracy (15–17).

### 2.3. Statistical analysis

SPSS 26.0 (IBM, Armonk, NY, USA) and Hiplot software (https://hiplot.com.cn/) were used for statistical analysis. The Kolmogorov-Smirnov method was used to test the normality of the continuous data. The continuous data conforming to the normal distribution were expressed as means ± standard deviations and compared using Student *t*-test. The continuous data conforming to skewed distribution were presented as medians (range), and compared using Mann-Whitney *U*-test. Categorical data were presented as *n* (%) and analyzed using Fisher's exact test. The bonferroni test was applied for multiple testing in pairwise comparing. Correlations between mitochondria parameters and serum bilirubin were examined using the ggscatterstats function in the “ggstatsplot” package (18), Spearman and Wilcoxon rank sum tests were performed to explore *P*. A two-sided *P *< 0.05 were considered statistically significant.

## 3. Results

**Figure 1:**
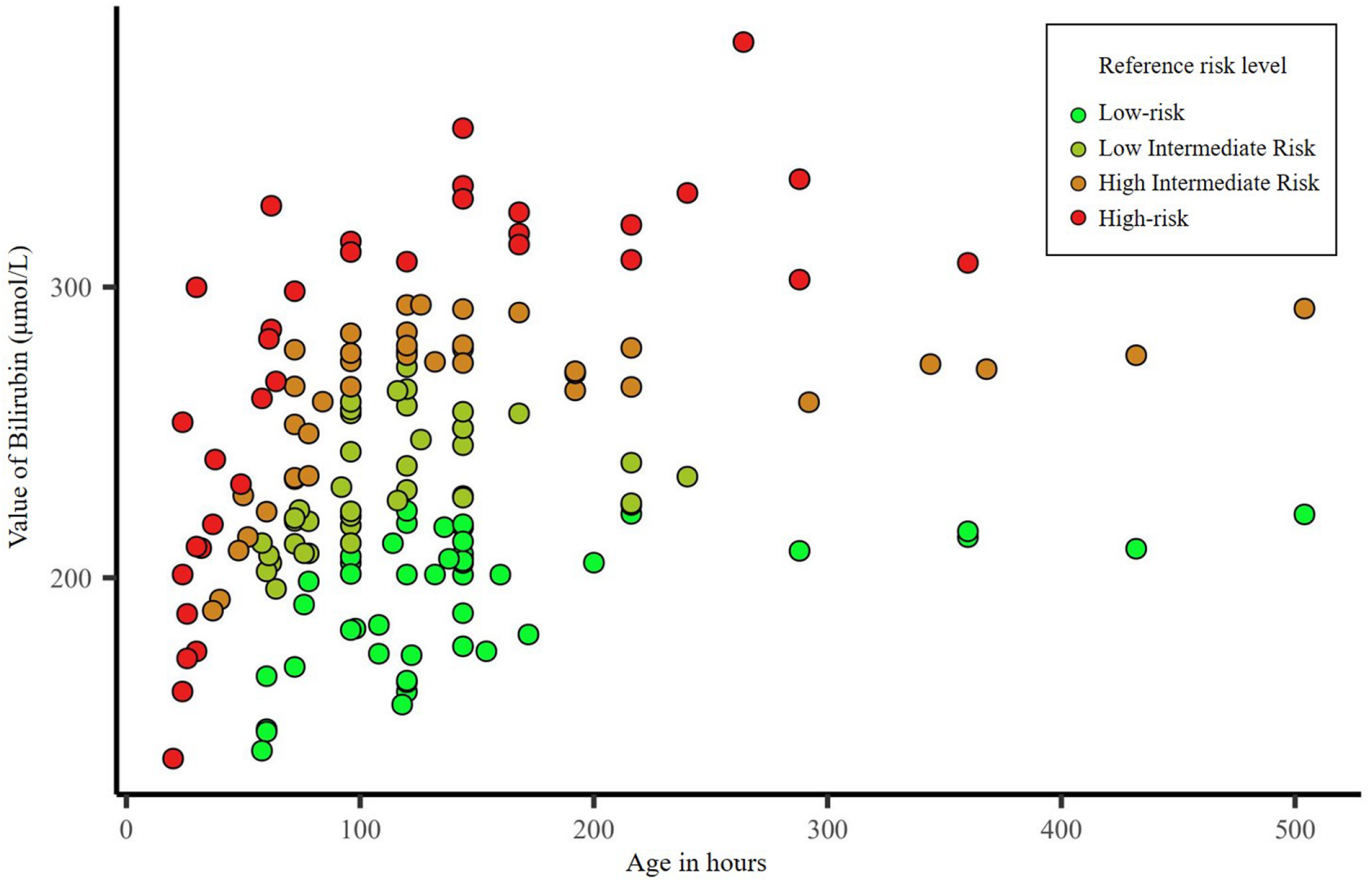
Distribution of serum bilirubin value according to hyperbilirubinemia risk.

There were 353 neonates with jaundice during the study period; 123 premature infants (gestational age <37 weeks) and 68 neonates with severe illness such as neonatal sepsis and intracranial hemorrhage were excluded. Therefore, 162 neonates (47, 41, 39, and 35 with low, intermediate-low, intermediate-high, and high-risk) were included in this study. There were no significant differences among the four groups regarding sex (*P *= 0.941), age (*P *= 0.091), gestational age (*P *= 0.100), and birth weight (*P *= 0.481) (Table 1). While the mean serum bilirubin value differed significantly among jaundiced neonates with different hyperbilirubinemia risks (*P *< 0.001). Figure 1 shows the distribution of the neonates' bilirubin values according to hyperbilirubinemia risk.

**Table 1:** Demographic and clinical characteristics of neonates with different risk levels of jaundice.

Characteristics	Low-risk (*n* = 47)	Low-intermediate-risk (*n* = 41)	High-intermediate-risk (*n* = 39)	High-risk (*n* = 35)	*P*
Sex (male/female)	23/24	24/17	21/18	17/18	0.941
Age (hours)	132.00 (98.00, 144.00)	96.00 (77.00, 144.00)	120.00 (72.00, 192.00)	64.00 (30.00, 168.00)	0.091
Gestational age (days)	276.00 ± 12.56	276.00 ± 8.71	277.00 ± 9.67	277.00 ± 9.55	0.100
Birth weight (g)	3,255.00 ± 317.60	3,290.00 ± 258.04	3,280.00 ± 226.88	3,390.00 ± 209.51	0.481
Serum bilirubin (μmol/L)	201.14 ± 22.72	228.13 ± 21.32	271.75 ± 27.72	299.90 ± 62.40	<0.001

The CD3^+^ and CD4^+^ T-cell percentages were lower in the high-intermediate and high-risk groups than in the low-risk groups (*P *< 0.0083). The CD4^+^/CD8^+^ ratio was lower in the high-risk group compared with the low-risk and intermediate-low-risk groups (*P *< 0.0083). Absolute counts of CD3^+^ cells were lower in the high-risk group compared with the low-risk group (*P *< 0.0083). Absolute counts of CD4^+^ cells were lower in the high-risk group compared with the low-risk and intermediate-low-risk groups (*P *< 0.0083). Besides, absolute counts of CD8^+^ cells have not significant difference (Table 2 and Supplementary Table S1).

**Table 2:** Comparison of cell percentages, absolute counts, and mitochondria-related parameters between neonates with different risk levels of jaundice.

Characteristics	Low-risk (*n* = 47)	Low-intermediate-risk (*n* = 41)	High-intermediate-risk (*n* = 39)	High-risk (*n* = 35)	*P*
CD3^+^ %	83.69 ± 13.31	76.85 ± 13.02	72.05 ± 11.22^a^	68.59 ± 10.85^a^	<0.001
CD4^+^ %	60.27 ± 10.64	57.58 ± 10.90	51.63 ± 9.01^a^	48.14 ± 11.22^a^	<0.001
CD8^+^ %	22.23 ± 10.18	19.25 ± 6.47	21.13 ± 9.02	23.28 ± 12.34	0.118
CD4^+^/CD8^+^	2.54 ± 1.18	2.78 ± 1.04	2.49 ± 0.91	2.02 ± 1.13^a^^,^^b^	0.002
CD3^+^ Abs. Count	2,658.37 (1,357.24, 4,610.70)	2,348.00 (1,585.25, 3,123.76)	2,196.00 (1,274.98, 2,933.98)	1,773.60 (1,093.85, 2,439.00)^a^	<0.001
CD4^+^ Abs. Count	1,880.00 (1,033.08, 3,393.49)	1,695.60 (1,185.25, 2,338.50)	1,604.82 (966.91, 2,234.00)	964.59 (683.31, 1,569.83)^a^^,^^b^	<0.001
CD8^+^ Abs. Count	753.67 (415.00, 988.56)	524.00 (386.77, 830.15)	547.04 (327.55, 930.14)	569.44 (358.15, 669.16)	0.010
CD3^+^ MM	2,29,646.80 ± 1,99,190.20	2,57,518.80 ± 2,06,475.39	2,61,932.00 ± 1,48,714.71	3,23,996.00 ± 1,71,894.44	0.280
CD4^+^ MM	2,40,010.40 ± 2,05,319.90	2,57,709.60 ± 1,80,679.68	2,98,482.00 ± 1,54,353.10	3,22,954.40 ± 1,87,833.49	0.359
CD8^+^ MM	2,39,636.00 ± 1,95,974.55	2,23,939.00 ± 1,94,679.45	2,59,279.20 ± 1,46,063.70	3,04,928.00 ± 1,74,845.57	0.343
CD3^+^ SCMM	61.39 ± 48.76	62.45 ± 42.00	76.30 ± 54.25	111.82 ± 98.44^a^^,^^b^	<0.001
CD4^+^ SCMM	86.65 ± 65.50	91.84 ± 55.71	106.79 ± 71.64	190.60 ± 146.38^a^^,^b^,^c^^	<0.001
CD8^+^ SCMM	198.01 ± 142.56	276.21 ± 173.61^a^	289.40 ± 118.29	299.97 ± 148.34^a^	<0.001

MM, mitochondrial mass; SCMM, single cell mitochondrial mass; Abs. count, absolute count.

^a^ *P *< 0.0083 compared to the low-risk group.

^b^ *P *< 0.0083 compared to the low-intermediate-risk group.

^c^ *P *< 0.0083 compared to the high-intermediate-risk group.

**Figure 2:**
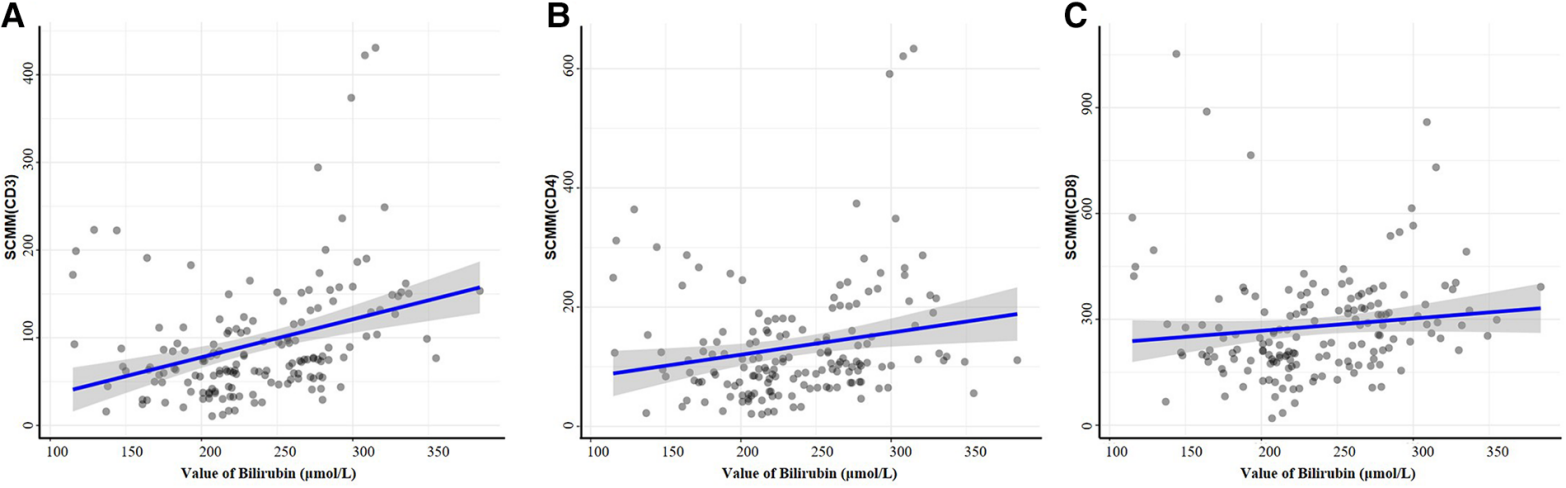
Correlations between bilirubin and SCMM parameters by the function ggscatterstats. Correlation analysis of peripheral serum CD3^+^ cell SCMM values (**A**), CD4^+^ cell SCMM values (**B**), CD8^+^ cell SCMM values (**C**), and peripheral bilirubin values.

There were no differences among the four groups regarding the MM parameters (all *P *> 0.05). The CD3^+^ SCMM was higher in the high-risk group compared with the low and intermediate-low-risk groups (both *P *< 0.0083), CD4^+^ SCMM was higher in the high-risk group compared with the three other groups (all *P *< 0.0083), and CD8^+^ SCMM was higher in the intermediate-low and high-risk groups compared with the low-risk group (both *P *< 0.0083) (Table 2 and Supplementary Table S1). CD3^+^ (*r* = 0.34, *P *< 0.001) and CD4^+^ (*r* = 0.20, *P *= 0.010) SCMM correlated with bilirubin levels positively (Figure 2).

## 4. Discussion

This study suggested that the mitochondrial CD3^+^, CD4^+^, and CD8^+^ SCMM parameters differed among jaundiced neonates with different hyperbilirubinemia risks. CD3^+^ and CD4^+^ T cell SCMM values were positively and linearly correlated with the serum bilirubin levels. The results might provide an organelle view for further studies on immunologic function and jaundice.

The process of bilirubin metabolism requires the direct involvement of the mitochondria (7, 9–11), oxidizing bilirubin into non-toxic, soluble biliverdin in the mitochondria and preventing toxicity from reactive oxygen species; biliverdin is then transported to the cytoplasm and reduced to bilirubin, which is subsequently released from the cytoplasm into the blood (19). In contrast, excess free bilirubin is highly toxic as it promotes oxidative stress and lipid peroxidation, leading to membrane damage and, ultimately, apoptosis (10, 20). Excess bilirubin can directly disrupt the membrane lipids, proteins, and redox status of mitochondria (21) and is toxic to the mitochondria (22). On the other hand, serum bilirubin concentrations below apoptotic levels affect cellular mitochondrial function and cellular activity by increasing the osmotic pressure of the mitochondrial matrix, causing it to break down and release apoptosis-inducing factor (AIF), leading to alterations of the cellular signaling pathways, affecting cellular activity, or ultimately initiating mitochondrial apoptotic mechanisms (20). Serum bilirubin has antioxidant and anti-inflammatory effects and often acts as an immunomodulator (23). Serum bilirubin has also been reported to promote the regeneration of Treg cells (24) and to reduce T cell numbers and activity by upregulating Treg cells for lymphocyte immunosuppression (25). Therefore, high serum bilirubin levels have a profound effect on immune cell activity and mitochondrial function and can leave the jaundiced neonate in an immunocompromised or immunosuppressed state, with a high risk of infection.

Indeed, in the present study, as the risk of clinically significant jaundice increased, the absolute immune cell count tended to decrease, and the CD3^+^, CD4^+^, and CD8^+^ SCMM parameters were significantly higher in the high-risk group than in the low-risk group, suggesting diminished immune cell function. CD3^+^ and CD4^+^ T cell SCMM values showed positive linear correlations with the serum bilirubin levels, suggesting that CD4^+^ T cells may be related to bilirubin via mitochondrial metabolism. The immune system of newborns is not well developed, and the mitochondrial metabolism capacity of immune cells can be insufficient to deal with the large accumulation of bilirubin. Hence, high bilirubin levels in jaundiced neonates might be associated with mitochondrial damage or dysfunction. On the other hand, a higher concentration of bilirubin would have a more significant suppressive effect on the body's immune system and a greater impact on cellular function impairment (10). Therefore, neonates with neonatal jaundice showed significant differences in relative and absolute immune cell counts and SCMM and showed linear correlations between SCMM and bilirubin levels across the different hyperbilirubinemia risks.

Serum bilirubin levels showed a positive linear correlation with CD3^+^ and CD4^+^ T cell SCMM but not with CD8^+^ T cell SCMM, suggesting that high blood bilirubin levels have a more significant effect on mitochondrial abnormalities/dysfunction in CD4^+^ T cells than in CD8^+^ T cells. These results were consistent with the study by Liu et al. (26), who reported that high bilirubin levels induced apoptosis in activated CD4^+^ cells and produced direct cytotoxicity. The effect of bilirubin on immune cells might be selective. Indeed, CD8^+^ cells appear to have higher SCMM values than CD3^+^ and CD4^+^ cells, which could be because CD8^+^ cells mainly rely on glycolysis for energy production (27, 28). Still, even though no correlations were observed between CD8^+^ SCMM and serum bilirubin levels, the CD8^+^ SCMM increases with the risk grouping.

This study has strengths. In this study, blood collection from neonates with jaundice was performed before phototherapy, and the effects of gestational age, age, and birth weight on bilirubin were excluded. This study also has limitations. The neonates were from a single center, and the sample size was small. There were limitations in the variability of data collection and recording. The outcomes of the neonates were not available. The results need to be confirmed in large multicenter studies.

In conclusion, the mitochondrial SCMM parameters differed significantly among jaundiced neonates with different hyperbilirubinemia risks. CD3^+^ and CD4^+^ T cell SCMM values were positively correlated with the serum bilirubin levels, and might correlated with hyperbilirubinemia risk. This study provides new insight into the effect of serum bilirubin on immune cell mitochondria, and the linear relationship between CD4^+^ cell SCMM and bilirubin may also open up new diagnostic ideas, thus improving the clinical understanding of immune cells in jaundiced neonates and providing a basis for the management of clinically relevant jaundice.

## Data Availability

The original contributions presented in the study are included in the article/**Supplementary Material**, further inquiries can be directed to the corresponding author.
