# Supplementary material for: Correlation between hyperbilirubinemia risk and immune cell mitochondria parameters in neonates with jaundice
Source: Front Pediatr. 2023 Jun 16;11:1200099. doi: 10.3389/fped.2023.1200099 (PMC10313225; doi:10.3389/fped.2023.1200099)
Supplement: Supplementary file 1 [file Table1.docx]

**Table 2.** Relevance according to the frequency of echo measurement in the selected studies

|  | **Reported parameter** | **Study^#^** | **Range limits** |
| --- | --- | --- | --- |
| **Right ventricle** | TAPSE | 12, 22, 23, 24 | Z score calculator for GA |
|  | TV A | 12, 16, 19, 20, 24, 25, 26 | 48.9 cm/sec in preterm to 54.9 cm/sec in term infants |
|  | TV E | 12, 16, 19, 20, 24, 25, 26 | 38.2 cm/sec in preterm to 47.9 cm/sec in term infants |
|  | TV E/A | 12, 14, 16, 19, 20, 24 ,25, 26 | 0.78 in preterm to 0.88 in term infants |
|  | RV E/e’ | 12, 16, 19 | 6.11±1.43 |
|  | RVO (within first 24 hours) | 10, 14, 15, 17 | 235 to 255 ±0.3 ml/kg/min |
|  | S’ -RV | 12, 16, 19, 20, 23, 26 | 6.2-6.6 cm/sec |
| **Pulmonary vascular resistance** | PAAT, RVET, PAAT: RVET | 14, 18 | PAAT= 33 to 35 ms  RVET=166 to 216 ms  PAAT: RVET= 5.1 to 4  PAPs =12 mmHg |
|  | Shunt at the PDA level | 15 |  |
|  | Estimated pulmonary artery pressure from TR jet (within first 24 hours) | 10, 25 |  |
|  | Interventricular septal flattening | 12 |  |
| **Left ventricle** | MV A | 13, 16, 19, 20, 21, 24,25, 26 | 43.2 cm/sec in preterm to 47 cm/sec in term infants |
|  | MV E | 13, 16, 19, 20, 21, 24, 25, 26 | 32.7 cm/sec in preterm to 61.5 cm/sec in term infants |
|  | MV E/A | 13, 16, 19, 20, 21, 24,25, 26 | <1 in preterm to ≤ 1 in term infants |
|  | LV E/e’ | 16, 19, 20 | 8.31±1.67 |
|  | LVO (within first 24 hours of age) | 10, 13, 14, 15, 19 | 138 to 143 ml/kg/min |
|  | LVSV | 14, 15, 17 | 3.3 to 4.2 ml/kg/min in the first 24 hours |
|  | EF Simpson | 13, 14, 17, 19, 20, 25 | 55-65% |
|  | SF | 13, 15, 19, 20, 21, 25, 26 | 28 - 40% |
|  | S’-LV lateral wall | 13, 14, 16, 19, 20, 23, 26 | 5.3 to 5.5 cm/sec |
|  | S’-interventricular septum | 13, 20, 23, 26 | 3.7-3.9 cm/sec |

^#^ Number refers to paper listed in References.

Abbreviations: TAPSE Tricuspid annular plane systolic excursion, TV Tricuspid valve, A peak atrial velocity, E peak early velocity, RV right ventricle, E peak early diastolic flow velocity, e’ peak early diastolic motion velocity, RVO right ventricle output, S’ peak systolic motion velocity, PAAT pulmonary artery acceleration time, RVET right ventricle ejection time, PDA patent ductus arteriosus, MV mitral valve, LV left ventricle, LVO left ventricle output, SV stroke volume, EF ejection fraction, SF shortening fraction
